# Disease mechanisms in preclinical rheumatoid arthritis: A narrative review

**DOI:** 10.3389/fmed.2022.689711

**Published:** 2022-08-19

**Authors:** Vasco C. Romão, João Eurico Fonseca

**Affiliations:** ^1^Rheumatology Department, Hospital de Santa Maria, Centro Hospitalar Universitário Lisboa Norte, Lisbon Academic Medical Centre and European Reference Network on Rare Connective Tissue and Musculoskeletal Diseases Network (ERN-ReCONNET), Lisbon, Portugal; ^2^Rheumatology Research Unit, Faculdade de Medicina, Instituto de Medicina Molecular João Lobo Antunes, Universidade de Lisboa, Lisbon, Portugal

**Keywords:** rheumatoid arthritis, Pre-RA, preclinical rheumatoid arthritis, pathogenesis, etiology

## Abstract

In the last decades, the concept of preclinical rheumatoid arthritis (RA) has become established. In fact, the discovery that disease mechanisms start years before the onset of clinical RA has been one of the major recent insights in the understanding of RA pathogenesis. In accordance with the complex nature of the disease, preclinical events extend over several sequential phases. In a genetically predisposed host, environmental factors will further increase susceptibility for incident RA. In the initial steps of preclinical disease, immune disturbance mechanisms take place outside the joint compartment, namely in mucosal surfaces, such as the lung, gums or gut. Herein, the persistent immunologic response to altered antigens will lead to breach of tolerance and trigger autoimmunity. In a second phase, the immune response matures and is amplified at a systemic level, with epitope spreading and widening of the autoantibody repertoire. Finally, the synovial and bone compartment are targeted by specific autoantibodies against modified antigens, initiating a local inflammatory response that will eventually culminate in clinically evident synovitis. In this review, we discuss the elaborate disease mechanisms in place during preclinical RA, providing a broad perspective in the light of current evidence.

## Introduction

The pathophysiology of rheumatoid arthritis (RA) is complex and yet incompletely understood (1, 2). Extensive epidemiological and etiological data emerging over the last decades have made a decisive contribution toward improving the knowledge of the mechanisms involved in the initiation and perpetuation of the disease (3). As demonstrated by the plethora of immune-related host and environmental risk factors for RA, the immune system as a whole plays a central role in the disease pathogenesis (3). It is therefore regarded as a prototypical immune-mediated disease (1, 4).

**Figure 1:**
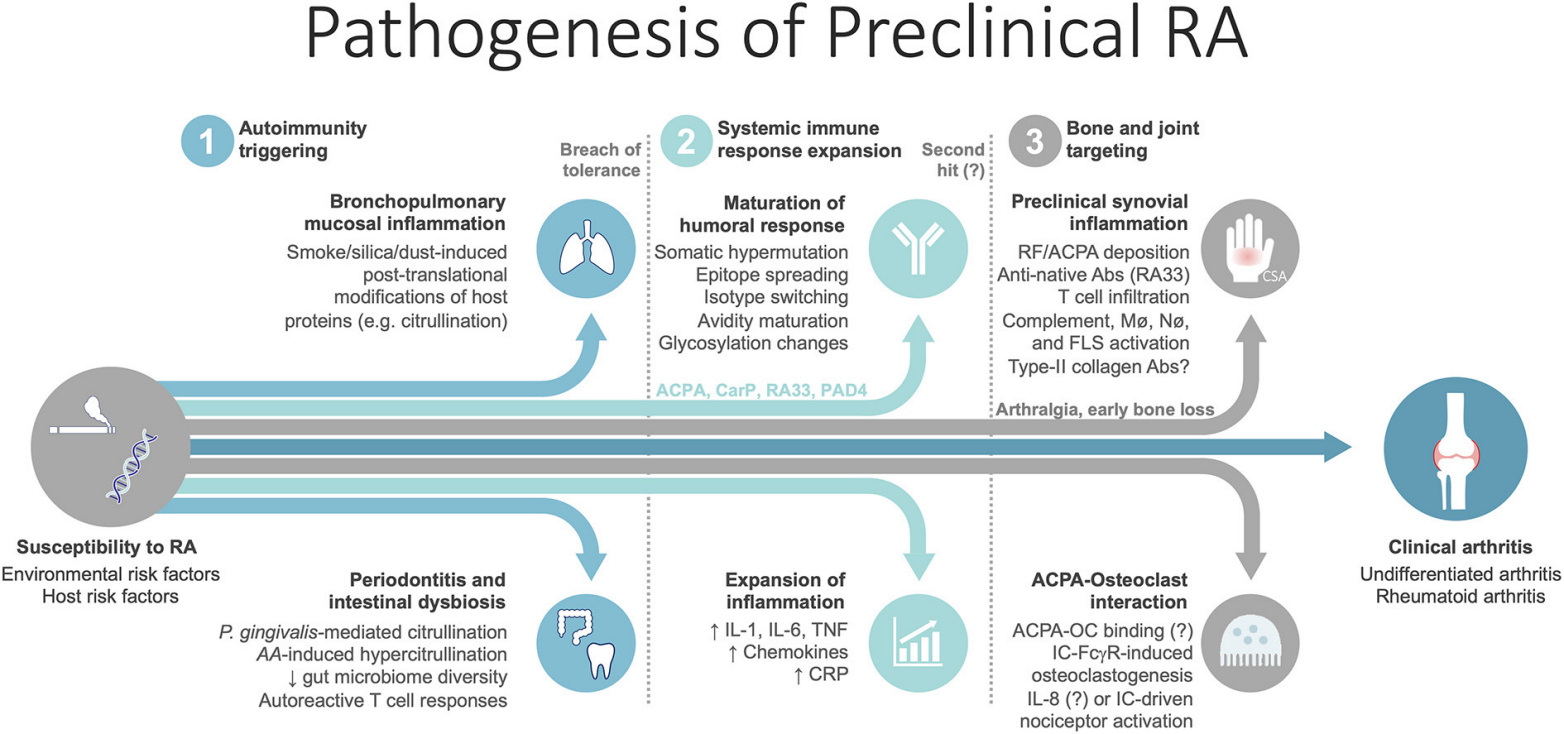
Summary of the pathogenic steps unfolding in the preclinical phase of rheumatoid arthritis. Mechanisms for which evidence is conflicting and significant uncertainty remains are followed by a question mark. *AA, Aggregatibacter actinomycetemcomitans*; Abs, antibodies; ACPA, anti-citrullinated protein antibodies; CarP, carbamylated protein; CSA, clinically suspect arthralgia; FLS, fibroblast-like synoviocytes; IC, immune complexes; Mø, macrophages; Nø, neutrophils; OC, osteoclast; *P. Gingivalis, Porphyromonas gingivalis*. PAD4, peptidyl-arginine deiminase 4; RA33, heterogeneous nuclear ribonucleoprotein (hnRNP)-A2, also known as RA33; RF, rheumatoid factor.

A comprehensive body of evidence emerging from the last two to three decades has generated many hypotheses, that currently regard RA as a continuum of consecutive phases that unfold longitudinally (Figure 1) (1, 4–7). The first phase corresponds to the general susceptibility to RA, when there are no symptoms or detectable ongoing immune disturbances. Herein, environmental stimuli interact with a genetically predisposed, epigenetically modified host to increase susceptibility to RA [reviewed in detail elsewhere (3)]. The result of these interactions in sites such as the mucosal surfaces (e.g., lung, gums, gut) may lead to the second phase—of preclinical RA—when breach of tolerance occurs and autoimmunity initiates. This seems to be followed by maturation and amplification of the autoimmune response outside the joint (e.g., in secondary lymphoid organs), whereby epitope spreading takes place and blood titers of autoantibodies (e.g., rheumatoid factor [RF], anti-citrullinated protein antibodies [ACPA] and others) increase. In parallel, systemic inflammation expands and levels of circulating cytokines and acute phase reactants rise (4, 6). In a proposed early intermediate step, joint and bone tissue may be targeted by the expanding ACPA repertoire, with minor immune cell infiltration (6, 8). During this phase patients are initially asymptomatic, but soon develop arthralgia and early bone loss (6, 8, 9). Following mostly undisclosed events, which might include a “second hit” (e.g., trauma, infection), elements of the activated adaptive and innate immune systems begin to access synovial tissue as local vascular permeability increases, resulting in overt synovitis that is clinically detectable (1, 6). Replication of this process in several joint sites culminates in the typical polyarticular phenotype of RA, enabling clinical diagnosis. Over time, complex interactions between innate and adaptive immune cells in the inflammatory synovium microenvironment, reinforced by positive feedback loops, synovial neoangiogenesis, insufficient lymphangiogenesis and apoptosis resistance, lead to perpetuation of synovitis (1, 2). A marked stromal tissue response involving fibroblast-like synoviocytes, chondrocytes and osteoclasts, further integrates with the ongoing inflammation to determine tissue reorganization and joint destruction (1, 2, 8). Recently, high-throughput analyses integrating cutting-edge techniques, such as single-cell RNA sequencing and mass cytometry, have shed light on the complexity of RA synovitis by identifying 18 cell populations within the synovial tissue of patients with established disease, including 4 fibroblast subsets, 4 monocyte subsets, 6 T cell subsets and 4 B cell subsets (10). In addition, 4 expanded cell states in the rheumatoid synovia were identified, involving specific subsets of sublining fibroblasts, pro-inflammatory monocytes, autoimmune-associated B cells and T follicular and T peripheral helper cells. These are exciting findings that have been reviewed elsewhere (11) and beyond the scope of the present article. Whether these cell populations and inflammatory cell states are also present in the preclinical or early stages of RA is currently unknown.

It should be highlighted that most of these mechanisms that have been suggested are valid and mostly applied to seropositive (i.e., RF and/or ACPA-positive) RA. There are a few suggestions of comparable phenomena in seronegative disease, but a deep comprehension of the driving forces behind this subtype is still illusive, and far from being a reality (4, 6). Therefore, herein, we will mainly focus on the pathogenesis of seropositive RA, and we will summarize what is currently known regarding seronegative disease.

The first pathophysiological phase, related to the genetic, epigenetic and environmental risk factors for the development of RA, has been addressed elsewhere (3) and is beyond the scope of this review. Herein, we focus on the events that occur during preclinical RA and eventually lead to early RA.

## Pathogenesis of preclinical RA

The concept that RA begins years before it is clinically evident has been one of the main advances in the understanding of the disease pathogenesis (4). This was first suggested by seminal work showing that RF (12, 13) and ACPAs (13, 14) often precede the development of clinical RA. Around half of the patients with RA have positive determinations of RF and/or ACPAs for a median of 4.5 years (and up to almost 14 years) before the onset of symptoms (14). Subsequently, ACPA positivity was also demonstrated to antedate and predict future development of arthritis in patients with arthralgia (hazard ratio [HR] 6.0, 95% confidence interval [CI] 1.8–19.8) (15). Similar findings were reported for other RA-related autoantibodies such as anti-carbamylated protein (anti-CarP) antibodies, which may be important in a proportion of ACPA-negative patients (16). Moreover, ACPA status is unlikely to change following the diagnosis, strengthening the hypothesis that these autoantibodies have a direct influence in the development of RA during the preclinical phase (17). Simultaneously, the genetic background of RA started to be dissected and the major contributions of the human leukocyte antigen (HLA) region and of the shared epitope (SE) were revealed, confirming the prominent role of antigen presentation to T cells and T cell-mediated immunity in the pathogenesis of the disease, as already evidenced by the presence of ACPAs (18–20). These studies provided support to a pathogenic model in which breach of tolerance occurs long before clinical disease, due to the interaction of genetic and non-genetic risk factors, and leads to an autoimmune response that is further propelled until RA surfaces. In this process, epigenetic regulation mechanisms are also at play in the interaction between the environment and the genetic background of the host. Indeed, they may contribute to the low concordance rate observed between monozygotic twins (9–15%) (21–23). A recent large epigenome-wide association study found differentially variable methylation signatures in monozygotic twin pairs discordant for RA (24). These altered DNA methylation patterns have been well document in RA patients, suggesting a possible pathogenic role (25–29). Interestingly, specific methylome signatures have even been described across different joints of the same patient, possibly contributing for the characteristic articular pattern of RA (30, 31). Histone modification is another epigenetic process that can influence gene expression. Although less studied, altered acetylation status have been identified in blood and synovial tissue of RA patients (32–35). Further, an interaction of smoking, a major environmental risk factor, with histone deacetylases has been recently described (36, 37). Finally, microRNAs and long non-coding RNAs have also been implicated as regulators of RA susceptibility and severity. microRNA-155 and micro-RNA146a are among the best studied, and have been shown to be increased in blood, synovial tissue/fluid, and specific cell types, with pleotropic effects that promote inflammation and bone erosion pathways (38–41).

### Mucosal triggering of autoimmunity

Considering the temporal lapse between immune dysregulation and articular disease onset, it arises that early events may be distinct from those in place during the synovitis phase. Indeed, triggering of autoimmunity is thought to take place outside the joint compartment through key interactions between the host and external stimuli, which are mediated by the immune system (6). Genetic and epidemiological studies supplied fundamental evidence implicating mucosal surfaces as relevant sites involved in this process, being the central interfaces between the outer environment and body defenses (1, 6, 42). The lung, gum and gastrointestinal mucosae have all been proposed as possible sites where early pathogenic events take place, summarized in Table 1 (6, 42, 82).

**Table 1:** Main findings and possible mechanisms supporting a mucosal triggering of autoimmunity in RA.

**Mucosal compartment**	**Key findings**	**References**
Lung	Smoke, dust, silica and air pollution exposure as key risk factors for RA	(43–48)
	Serum IgA ACPA/RF and IgA-plasmablasts enriched in preclinical RA	(13, 49)
	Sputum IgA/IgG ACPAs enriched in the sputum of early RA patients and at-risk individuals	(50, 51)
	Airway/parenchymal inflammation in RF/ACPA+ asymptomatic individuals and ACPA+ RA	(52, 53)
	Smoke-induced Toll-like receptor triggering, PAD2 upregulation and citrullination	(54, 55)
	Higher lung ACPA concentration (vs. serum), induced BALT with ELS, PAD2 expression and citrullination	(53, 56)
Gum	Association of periodontitis with prevalent (and incident) RA	(57–60)
	*P. gingivalis* and PPAD citrullinate RA-specific autoantigens, targeted by ACPAs in preclinical/early RA	(61–63)
	Periodontal PAD activity, protein citrullination, and ACPA responses in patients with periodontitis	(64–67)
	Higher prevalence/severity of periodontitis in ACPA+ FDR (vs. ACPA-)	(68)
	Molecular mimicry/cross reactivity of ACPAs—anti-*P. gingivalis* antibodies in at-risk and RA populations	(69–72)
	PPAD autocitrullination (?) and anti-native PPAD antibodies	(73–76)
	Increased *A. actinomycetemcomitans* frequency in early and (but not preclinical) established RA *Aa*-induced leukotoxic hypercitrullination—ACPA-targeting; overlapping periodontal-synovial citrullinome	(67, 77–81) (67)
	Changes in oral and subgingival microbiota in untreated RA, partially normalized by csDMARDs	(82–84)
	Non-surgical periodontal treatment improves RA disease activity	(85–87)
Gut	Gut microbiome altered in patients with RA (expanded *Prevotella copri*)	(82, 88–92)
	Enrichement of *Prevotella* spp. in pre-RA subjects (ACPA/RF+ and/or CSA)	(93)
	Intestinal dysbiosis and cross-reactivity with gut bacterial peptides triggers RA-specific T cell responses	(91, 92)
	Molecular mimicry of bacterial proteins (*Prevotella* spp.) and RA-specific synovial-expressed autoantigens	(94)

*Aa, Aggregatibacter actinomycetemcomitans; ACPA, anti-citrullinated protein antibodies; BALT, bronchus-associated lymphoid tissue; CSA, clinically suspect arthralgia; csDMARDs, conventional synthetic disease-modifying anti-rheumatic drugs; ELS, ectopic lymphoid structures; FDR, first-degree relatives; PAD, peptidyl-arginine deiminase; PPAD, Porphyromonas gingivalis peptidyl-arginine deiminase; RF, rheumatoid factor*.

In this setting, it has been hypothesized that infectious agents, microbiota, cigarette smoke and other stressors interact with resident stromal and immune cells and initiate the chain of events that will eventually culminate in RA-specific autoimmunity. In fact, immunoglobulin (Ig)A-antibodies (ACPAs, RF) and IgA-plasmablasts are enriched in preclinical RA and predict evolution to overt disease, supporting the possible participation of mucosal immunity since the early pathophysiological stages (6, 13, 49).

It should be stressed, however, that the role of mucosal surfaces in initiating the pathogenic process has not been definitely proven and conclusive experimental evidence is lacking. It is likely that different mucosal compartments play different roles in separate patients, with the varying contribution of each surface, according to genetic, epigenetic and environmental factors. It is even possible that in some patients, mucosae play no role in the offset of autoimmunity and breach of tolerance. Thus, much remains to be determined and the content presented here is more appropriately seen as a working theoretical model.

#### Citrullination in RA

Given its prominent involvement in the pathogenesis of seropositive RA, a brief overview of citrullination is warranted. Citrullination is a physiological process that consists of the post-translational modification of arginine residues into the nonstandard amino acid citrulline. This reaction if catalyzed by calcium-dependent peptidyl-arginine deiminase (PAD) enzymes. Although PADs have been implicated in the pathogenesis of RA, they have multiple physiological roles, from epigenetic regulation to tissue differentiation (e.g., PAD1 in skin keratinization) (95). There are five isoforms of human PAD (PAD1-4 and PAD6), all widely expressed in multiple organs and systems, and each with a specific function [reviewed in detail elsewhere (95–98)]. The most relevant for RA are PAD2 and PAD4, both of which are expressed in immune cells (96). In particular, PAD4 is primarily expressed by neutrophils, eosinophils, and monocytes and is involved in epigenetic regulation through citrullination of histones and transcription factors (97–102). Both PAD2 and PAD4 can generate neoantigens, by catalyzing the citrullination of native widely expressed proteins such as fibrinogen or vimentin (95, 96, 103). Yet, they seem to have partially different targets and patterns of citrullination (95, 96, 103). More specifically, within the articular compartment, PAD4 may be more important for autoantibody generation, whereas PAD2 is more strongly linked to inflammation (96).

In addition to their role in citrullination, PADs have also been proposed to contribute to RA pathogenesis *via* other mechanisms. Animal studies have suggested both PAD2 and PAD4 enhance the immune response, as their absence leads to reduced antibody production, complement deposition and pro-inflammatory cytokine production, in parallel with skewing of T cell polarization (104–107). This amplifies clinical inflammation and disease severity (96). On the other side, PADs can themselves be targeted by the immune response. Numerous studies have described an increased frequency of anti-PAD antibodies in patients with RA (96, 108–110). Anti-PAD4 antibodies are the most studied, having been described in 22–45% of patients with established RA (96, 108, 109). They are highly specific for RA and associated with the presence of ACPAs, albeit being also detected in 2–19% of patients with seronegative RA (96, 109, 111). In addition, they are associated with disease activity and severity, as well as joint damage and radiographic progression (96, 108–110, 112). Their implication in RA development has been strengthened by the demonstration of their presence in a subset of patients with preclinical disease (113). Anti-PAD2 and anti-PAD3/4 antibodies have also been described in patients with RA (96, 114, 115). Among other mechanisms, it has been hypothesized that anti-PAD antibodies could be generated by exposure of high quantities of extracellular PAD enzymes to autoreactive B/T cells in a setting of intense inflammation, such as within the joint compartment (96, 116).

The implication of citrullination in the pathogenesis of RA has been a matter of debate over recent years (98). Neutrophil extracellular traps (NETs) formation following controlled neutrophil death (also known as NETosis) is an antimicrobial mechanism that has been widely proposed as a source of citrullinated antigens in RA *via* hypercitrullination (117–119). On the contrary, evidence shows that PAD4 activity actually decreases during NETosis, which is characterized by limited citrullination (e.g., of histone H3) and absence of hypercitrullination (98, 120). Therefore, it is unlikely that NETosis contributes in a significant fashion to the generation of citrullinated autoantigens that characterizes the early stages of RA pathogenesis. Instead, a separate mechanism that may be of relevance in this regard is another form of neutrophil death denominated leukotoxic hypercitrullination (LTH). It is a bacterial-induced immune-evading strategy that features a marked rise in intracellular calcium, resulting in PAD hyperactivation (namely PAD4), cellular protein hypercitrullination, and extrusion of DNA-containing NET-like structures (98, 120–123). The exposed citrullinated residues could then be targeted by the adaptive immune-system, mounting an autoimmune response that eventually leads to the development of RA.

#### The lungs and breach of tolerance

The idea that lungs may be a site in which autoimmunity is generated in some patients is supported by a variety of studies associating smoking and other airborne noxious agents, such as silica and occupational dust, with both RA and ACPA generation (3, 6, 42–46). These exposures were shown to interact with genetic markers (e.g., human HLA-SE) to multiply the risk, thereby suggesting a common pathogenic pathway (42, 47, 48). Moreover, a study reported that 76% of RF/ACPA-positive individuals without arthritis had airway and parenchymal inflammatory abnormalities on high-resolution computed tomography (HRCT), which were similar in type and frequency to those seen in early RA (52). On the other hand, early untreated ACPA-positive RA patients have been shown to have a higher rate of HRCT parenchymal lung changes and of bronchial biopsy microscopic inflammatory infiltrates than those with ACPA-negative RA and healthy controls (53). These data suggest that, in some patients, bronchopulmonary inflammation precedes the onset of synovitis and may possibly play a causal role in triggering RA-inducing autoimmunity.

The mechanisms through which smoking (and other inhaled agents) lead to loss of tolerance is not entirely comprehended. It has been proposed to involve post-translational modifications of common host proteins (1, 6). Citrullination, described above, is the best studied of those changes, but carbamylation (conversion of lysine to homocitrulline), acetylation and others have also been described (4). Smoke, silica or microbes can trigger Toll-like receptors (TLRs; e.g., TLR4) of dendritic cells, B cells and macrophages and increase expression and activation of PAD (6, 54, 55). In fact, smoking has been demonstrated to upregulate expression of PAD2 and citrullinated proteins in the bronchoalveolar lavage (BAL) and bronchial mucosal biopsies of smokers in comparison to non-smokers (42, 54).

However, citrullination *per se* is not sufficient to spawn autoimmunity as it occurs in the lungs of healthy smokers who do not develop RA and may even serve a physiological role in other tissues (4). Therefore, other factors must be present for loss of tolerance to citrullinated epitopes to occur. Notably, they include HLA molecules, particularly those carrying the SE sequence, which are particularly efficient at presenting citrullinated peptides to T cells (42, 124). Presentation of these self-neoantigens by HLA-SE-positive antigen presenting cells (APCs) leads to the emergence of autoreactive T cells, that interact with and provide help to B cells, resulting in the production of autoantibodies (i.e., ACPAs) and RF (targeting other antibodies) (1, 6). This hypothesis may explain the observation of the multiplicative risk of RA conferred by the coexistence of smoking and the SE (e.g., RR 21.0/1.5 in ever smokers carrying two/no copies of SE genes, respectively) (42).

Simultaneously, B cells attracted to the lungs by smoke substances can undergo local differentiation and recognize modified self-antigens (6). These may then be presented to autoreactive T cells in the context of germinal center-positive ectopic lymphoid structures (ELS) within the induced bronchus-associated lymphoid tissue (6). Whether this latter step is the result of defective thymic regulation of T cell clones recognizing post-translationally modified self-proteins is still unclear.

Several studies have provided circumstantial evidence of a possible involvement of the bronchopulmonary mucosal tissue in the loss of tolerance events that occur in RA. In this regard, a number of relevant observations have been reported: (i) IgA and IgG ACPAs are present in the sputum of early RA patients and enriched in that of at-risk individuals (including seronegative subjects) (50, 51); (ii) in at-risk subjects, ACPAs are associated with markers of local inflammation and NET formation, which can result in further liberation of citrullinated proteins and PAD4 (51); (iii) ACPAs are seen at higher concentrations in the BAL (vs. serum) of patients with early and established disease (53, 56); (iv) infiltration by activated lymphocytes and citrulline-specific plasma cells (e.g., induced bronchus-associated lymphoid tissue with numerous ELS), increased local expression of PAD2, and large areas of citrullinated proteins are commonly found in pulmonary biopsies of patients with early and established ACPA-positive RA (53, 56, 125).

The targets of the pulmonary ACPA response have also been studied. One of the aspects that puzzled investigators at first, but that is currently accepted, is that most ACPAs are directed at citrullinated proteins that are not specific to the synovium, but rather are ubiquitous in many other tissues alike (4–6). These include both intracellular histones and stromal proteins, such as vimentin, fibrinogen, fibronectin, α-enolase, tenascin C or type II collagen (1, 4, 6, 69). The specificities of ACPAs found in the sputum of at-risk individuals, recently reported, frequently included citrullinated forms of fibrinogen, vimentin, fibronectin and apolipoproteins E and A1, as well as non-citrullinated/native forms of some of these proteins (126). Interestingly, the autoantibody pattern of at-risk subjects differed from that of established RA patients, who frequently had ACPAs targeting histones and filaggrin, but also fibronectin and fibrinogen (126). Citrullinated fibrinogen in close relation with plasma cells has also been described in bronchial biopsies of established RA patients (56). These studies confirm that ACPAs generated in the respiratory mucosa match those that have been described in the serum of RA patients and that are commonly detected using commercially available techniques. Recently, five citrullinated peptides of some of these proteins (vimentin [*n* = 2], annexin A2, histone and actin) were reported to be shared between synovial and bronchial tissue of patients with RA, who also had circulating ACPAs against these targets (127). This is in accordance with previous identification of citrullinated antigens in the rheumatoid joint compartment and strengthens the hypothesis that RA-specific autoimmunity may begin in the lung.

Other parallel or complementary pathogenic mechanisms might possibly be involved since the early phases of immune dysregulation. In fact, it has been suggested that smoke-induced inflammation in the respiratory tree, rather than smoke *per se*, is responsible for peptide citrullination (128), and that this process is abundant in many tissues and driven by inflammation (129). Moreover, patients with bronchiectasis were shown to have increased rates of serum RF and anti-fibrinogen ACPAs, but the immune response was not citrulline-specific and also targeted native peptides (130). This contrasted with the highly specific ACPA response in RA patients who also had bronchiectasis (130). These findings indicate that chronic bacterial infection associated with bronchiectasis may contribute to induction of autoimmunity that becomes RA-specific following the initial loss of tolerance.

In accordance with this observation, a role for microbial infection and lung mucosal dysbiosis has been proposed. Smoking has been associated with increased bacterial colonization and modification of the lung microbiome, thereby proposing an additional route through which smoke can trigger RA-targeted autoimmunity (131). Moreover, changes in microbiota composition have been demonstrated in sputum samples of ACPA-positive at-risk arthritis-free subjects and in the BAL of early untreated RA patients (132, 133). Distal airway dysbiosis in RA was characterized by a reduction in both diversity and abundance of microbiota, some of which correlated with local/systemic levels of autoantibodies and disease activity (133). Intriguingly, the genera *Prevotella* and *Porphyromonas*, which have been linked to RA in other mucosal surfaces, were not increased in the lungs of early RA patients and were even found to be significantly reduced in comparison to healthy controls (133).

It should be noted that although these studies provide a basis to associate the lung with the loss of tolerance mechanisms that unfold in the preclinical stages, they do not establish any direct mechanistic link between smoking and citrullination in the pulmonary mucosal surface. In fact, current evidence on ACPA and PAD lung expression merely suggests, by association, that the lungs may contribute to the early breach of tolerance mechanisms, but is insufficient to establish this mucosal surface as the primary site of RA initiation.

#### The role of gingival and intestinal dysbiosis

Although there is data supporting lungs as an important site for breach of tolerance to occur, many patients with seropositive disease do not present any pulmonary changes or risk factors. Therefore, within the same framework of mucosal-driven inflammation, other sites have been proposed, most notably the gingival and intestinal mucosae (1, 6, 57, 134). Extensive epidemiological evidence implicates microbiota and external infectious agents with the development of RA and other immune-mediated diseases [reviewed in detail elsewhere (3, 134–138)]. On the other hand, microbiota itself changes throughout the course of disease, raising the question of whether some of the reported disturbances are actually a consequence, rather than a cause, of the disease (138). Nevertheless, there is considerable data implicating specifically intestinal and oral dysbiosis in the etiology of RA. In addition, important mechanistic experimental data is available for these compartments, providing support for the role of oral and intestinal mucosae in preclinical disease.

Periodontitis, results from dysbiosis of the gingival microbiota and is strictly associated with established RA, although the association with incident RA is somewhat inconsistent (57–60). This condition entangles mucosal dysbiosis and various pathogenic agents such as *Porphyromonas gingivalis* (*P. gingivalis*), which has the ability to citrullinate peptides through its own PAD (PPAD) (139, 140). Further, PPAD can provide a competitive advantage for *P. gingivalis*, protecting it from the natural acidification of the oral environment through the ammonia generated in the citrullination process (140, 141). There are, however, several differences between PPAD and the human PADs described above, in that the former can only citrullinate C-terminal arginine residues of autoantigens following its cleavage by arginine gingipains (61, 142). In addition, PPAD has the ability to citrullinate free L-arginine residues and does not require calcium ions for catalysis (140, 143). Importantly, experimental data demonstrate that PPAD can citrullinate several RA-specific autoantigens such as α-enolase, vimentin or fibrinogen (61–63). Of note, antibodies against PPAD-citrullinated vimentin were recently reported in patients with preclinical and early RA (63).

Studies have demonstrated periodontal PAD activity and protein citrullination, as well as local and systemic ACPA responses in patients with periodontitis (64–67). Recently, ACPA-positive first-degree relatives of RA patients were found to have a higher prevalence and severity of periodontitis than ACPA-negative subjects (68). Moreover, signs of molecular mimicry and cross reactivity between ACPAs and anti-*P. gingivalis* antibodies were shown in all stages of disease, from at-risk individuals to established RA (69–72). Such antibodies had additive interactions with other genetic (HLA-SE) and environmental (smoking) risk factors (70). These aspects fit well with the proposed lung-centered hypothesis explained above, whereby inflammation-induced and *P. gingivalis*-mediated citrullination occur at the gum and lead to loss of tolerance and ACPA generation.

Nevertheless, several aspects are a topic of controversy, and significant advances have been made in the last years. In particular, PPAD autocitrullination has been proposed as a possible mechanism of RA-specific autoimmunity, with conflicting results (73–76). Quirke et al. reported that a PPAD-mediated autocitrullination could elicit a specific immune response against citrullinated PPAD, which was demonstrated in the serum of a proportion of RA patients (73, 75). Subsequently, Konig et al. demonstrated that PPAD from *P. gingivalis* was not citrullinated, and was targeted by anti-PPAD antibodies in its native, but not citrullinated, form (74, 76). In addition, anti-PPAD antibodies were not associated with disease activity or ACPA titres in RA patients, and were even decreased in patients with coexisting RA and periodontitis (74). Contrarily, however, a recent study demonstrated that PPAD autocitrullination can occur *in vitro*, and antibodies against autocitrullinated PPAD can be detected in the serum of patients with preclinical, early or established RA (63). As such, despite the progress, this remains an open area of ongoing discussion.

Besides *P. gingivalis*, other gum-based pathways have been advanced. Exposure to *Aggregatibacter actinomycetemcomitans* (*A. actinomycetemcomitans*), a Gram-negative periodontitis agent, has been reported at increased levels in different independent cohorts of early and established RA patients, as translated by the presence of antibodies against bacterial proteins or DNA probes (67, 77–79, 142). Of note, in one of such studies, *A. actinomycetemcomitans* was the only periodontal pathogen that differed significantly between patients with or without RA, regardless of periodontitis status (78). As for preclinical RA, two recent studies failed to show an association between IgG against *A. actinomycetemcomitans* and presymptomatic stages of the disease (80, 81). Nonetheless, recent exposure to this pathogen, as reflected by IgM anti-leukotoxin A antibodies, may be associated with the development of clinical disease in a proportion of patients (80).

In addition to clinical data, there is also mechanistic evidence supporting the implication of *A. actinomycetemcomitans* in the pathogenesis of RA. In a recent study, Konig et al. elegantly showed that *A. actinomycetemcomitans* induces dysregulated activation of neutrophil PADs, through the action of the pore-forming leukotoxin A, mimicking membranolytic mechanisms that occur in the RA joint (67). This results in cellular hypercitrullination (LTH, as described above) that is followed by cytolysis and extracellular release of citrullinated proteins. These autoantigens can then be targeted by ACPAs and, indeed, anti-leukotoxin A antibodies were associated with periodontitis, RA and ACPA and RF-positivity (67). Importantly, the periodontitis citrullinome paralleled that of RA synovial fluid, including several major RA-specific citrullinated neoantigens that are targeted by ACPAs (e.g., vimentin, actin, α-enolase, histones and others), reinforcing the link between the inflamed gingival mucosa and the rheumatoid joint (67). Moreover, the spectrum of citrullinated proteins present and targeted in RA could be generated by *A. actinomycetemcomitans*, but not *P. gingivalis*. This interesting study provides a complex mechanistic description of an alternative route through which oral bacteria can trigger RA autoimmunity.

Although Engström et al. have recently suggested that local citrullination and PAD activation induced by chronic gingival inflammation were independent of periodontal pathogens such as *P. gingivalis* and *A. actinomycetemcomitans* (66), oral dysbiosis does seem to contribute to the genesis of RA. Differences in the oral microbiome have been described in patients with treatment-naïve RA (area under the curve [AUC] 0.87), that are partially normalized following conventional synthetic disease-modifying antirheumatic drug (csDMARDs) treatment (82). Likewise, subgingival microbiota of patients with new-onset RA differs from that of healthy controls and is characterized by the presence and/or abundance of species such as *Anaeroglobus geminatus* (associated with ACPA/RF-positivity), *Prevotella* and *Leptotrichia* (83). Moreover, an interesting study recently assessed the subgingival microbiome of RA patients without periodontitis and found it to be significantly different from controls, with similar frequencies of *P. gingivalis* and *A. actinomycetemcomitans*, but enrichment of proinflammatory gram-negative anaerobes and of citrulline-producing *Cryptobacterium curtum* (84).

Perhaps some of the most compelling data for a specific role of periodontitis in RA pathogenesis comes from studies evaluating the impact of non-surgical periodontal treatment in RA disease activity. A recent meta-analysis including 9 controlled studies of patients with concomitant RA and periodontitis concluded that non-surgical periodontal treatment resulted in clinical disease activity parameters such as DAS28, swollen and tender joint counts, patient global assessment and C-reactive protein (85). Two other subsequent clinical trials also confirmed these findings (86, 87). On the contrary, an interesting large study including over 400 patients with established RA and low disease activity, 56% of whom with periodontitis, failed to demonstrate a benefit of an oral hygiene intervention in improving disease activity, but did show a reduction of pathogenic bacteria load, including *P. gingivalis* (144). However, it should be stressed that baseline disease activity was low (mean DAS28 2.7 ± 1.3) and therefore less amenable to improvement, especially in patients with long standing disease (mean disease duration 9.0 ± 0.7 years), in whom remission is often a target difficult to attain. Furthermore, patients with baseline periodontitis seemed to have a greater benefit from oral hygiene measures, reinforcing the importance of periodontal health in the management of RA patients (144).

Finally, the intestinal mucosa has also been put forward as a possible site of early immune dysregulation in RA pathogenesis. The gut has a relevant contribution for the development and maintenance of the immune system, particularly through the interaction with commensal and foreign microbes at the mucosal surface (145). The importance of the intestinal microbiota composition as a determinant of human disease has recently been acknowledged, including in RA (134, 145).

Various studies have shown that the gut microbiome is altered in RA patients, with decreased diversity and expansion of several agents, most notably *Prevotella copri* (82, 88–92). Recently, *Prevotella* spp. has been demonstrated to be enriched in stool samples of pre-RA subjects (i.e., ACPA/RF-positive and/or suggestive RA signs/symptoms) in comparison with asymptomatic autoantibody-negative first-degree relatives of RA patients (93). While cross-sectional, these data support a role for intestinal dysbiosis since the early phases of RA development and should be confirmed by longitudinal studies.

Mechanistic studies reinforced this theory by demonstrating that autoreactive RA-specific T cell responses can be triggered by gut dysbiosis and cross-reactivity with intestinal bacterial peptides (91, 92). Furthermore, HLA-DR-presented, RA-specific autoantigens that are highly expressed in the synovial tissue have been shown to possess marked sequence homology with bacterial proteins of *Prevotella* spp. and other gut bacteria, suggesting a molecular mimicry process (94). T cell responses against the corresponding self and microbial peptides were correlated, providing yet another link between mucosal immunity and joint inflammation (94).

### Maturation and amplification of the systemic immune response

Following loss of tolerance and generation of specific autoantibodies, diverse mechanisms likely carry the pathogenic cascade forward through amplification of systemic autoimmunity (146). Firstly, epitope spreading occurs, during which more antigens are targeted by the immune response (6). While no particular single target has been found to determine the transition from the preclinical to the clinical stage, individuals who develop a wider variety of ACPAs and ACPA-specificities are more likely to progress to clinically apparent RA (13, 147–150). In addition to the broad autoantibody repertoire, ACPA titers (13, 148, 150) and, to a lesser extent, avidity (151) also rise progressively until the onset of disease. Moreover, subjects who go on to develop RA have a more diverse pattern of ACPA isotypes and subclasses (152).

Interestingly, there seems to be an uncoupling of the class-switch recombination and avidity maturation processes of B cell development. Extensive isotype switching is accompanied by only a relatively modest increase in ACPA avidity, which is significantly lower than that observed in other humoral responses against recall antigens (e.g., vaccination) (153). Nonetheless, despite its overall low levels, ACPA avidity maturation does occur during the preclinical phase until disease onset (151). Moreover, once RA becomes evident there does not seem to be a major subsequent variation of the recognition pattern, titer or avidity of ACPAs (149–151). Together, these data suggest that the maturation of the ACPA response occurs before the onset of clinical disease and, most likely, takes place outside the joint, in locations such as secondary lymphoid organs (e.g., lymph nodes), where high-affinity interactions take place between B and T cells (1, 6).

#### Systemic adaptive immune response

Evidence arising from large twin studies suggests that while environmental (e.g., smoking) and other random events are fundamental for the primary break of tolerance in RA autoimmunity, genetic factors such as HLA-SE are more important in the progression of the immune response described above and in the transition to immune-mediated disease (154). Adaptive immunity, therefore, is at the core of the maturation and amplification of systemic autoimmunity in pre-clinical RA.

Extensive literature directly implicates HLA molecules and their genetic variants as major determinants of the risk of RA (3, 18–20). These observations provide important indirect data for the pathogenesis of the disease, since antigen presentation to T cells is likely to be critical in the process (20). Subsequent studies have detailed the precise mechanisms through which specific amino acids located in the HLA-DR peptide binding groove (positions 11/13, 71, 74) create a positively charged pocket that accommodates neutral citrulline residues – but not positive arginine – of proteins such as vimentin, aggrecan, α-enolase or fibrinogen (6, 124, 155). This explains how post-translational protein modifications may lead to RA in a favorable genetic landscape, by facilitating the presentation of autoantigens by the HLA-DR molecule to T cells, *via* interaction with the T cell receptor (TCR). Indeed, autoreactive CD4^+^ T cells specific for citrullinated vimentin and aggrecan were shown to be present in RA patients carrying the risk allele HLA-DRB1^*^04:01 and its numbers were correlated with disease activity parameters (124). Moreover, the most important genetic risk variant outside the HLA region is the R620W single nucleotide polymorphism (SNP) in the *PTPN22* gene, which changes the activation threshold of T cells (and also B cells, to a lesser extent), resulting in the escape from thymic negative selection and in the generation of autoreactive cells (156). These data clearly support a role for T cells since the early stages of the immune response, which react to neoantigens presented by APCs and provide decisive help to B cells in the production of autoantibodies against several targets (e.g., ACPAs).

In this regard, the importance of a novel special subpopulation of T cells in the generation of antibodies and autoantibodies has been revealed in the last two decades. These T cells express an important surface chemokine receptor – CXC-chemokine receptor 5 (CXCR5) – that enables them to access the B cell follicle to provide fundamental help to B cells and were, therefore, named T follicular helper (Tfh) cells (157). More specifically, Tfh cells are instrumental in establishing and driving the germinal center response in secondary lymphoid organs, during which, with their help, B cells undergo clonal expansion, somatic hypermutation and affinity maturation, essential steps in an effective humoral response (157, 158). These functions are balanced by their subsequently described counterparts, T follicular regulatory (Tfr) cells, which possess suppressive capacities (159, 160). Since their discovery, both of these subsets have been implicated in the pathogenesis of RA and other immune-mediated rheumatic diseases characterized by autoantibody production and ELS formation (161). However, studies in RA have, so far, been mostly limited to demonstrating either a role for Tfh cells in the initiation and progression of disease in animal models of autoimmune arthritis (162, 163), or altered frequencies of circulating Tfh and Tfr populations in patients with early or established RA (164–166). Therefore, the disturbances and influence of these cell populations during the preclinical stages of RA, while appealing and of great potential, remain to be confirmed and definitely merit further study.

The implication of B cells in the preclinical stages of RA arises mostly from the wealth of studies (detailed above) demonstrating the complex dynamics of autoantibody production, as a surrogate of autoreactive B cell responses (146). Although earlier studies were unable to directly study B cells, these have been clearly established as major contributors to disease pathogenesis by different mechanisms (167). Besides their role in pathogenic autoantibody production (e.g., RF and ACPA), B cells can serve as potent APCs (presenting self-antigens and activating T cells) (168), can produce proinflammatory cytokines (169), and are critical for ELS formation (167, 170).

Disturbances in circulating B cell subpopulations have been identified since the very early stages (<6 weeks) of RA (171), alongside a cytokine pattern that favors B cell survival and activation (172, 173). Moreover, central and peripheral B cell tolerance checkpoints seem to be impaired, as the frequency of autoreactive B cell clones is increased in early and established RA patients (174). Similarly to T cells, this may be related to SNPs in *PTPN22* that affect B cell receptor signaling thresholds and lead to the emergence of mature B cells capable of recognizing citrullinated antigens (146, 156).

Indeed, methodological advances have recently enabled to identify the presence of citrullinated antigen-specific B cells (i.e., ACPA-expressing, IgG or IgA) (175–177), plasmablasts (178–180), and plasma cells (181) at relatively high levels in the peripheral blood of RA patients. These ACPAs are notable for their broad multi-reactivity, targeting an expanded set of citrullinated antigens, including those located at the joints (e.g., α-enolase or fibrinogen) (176–181). Additionally, they were found to directly promote the development of arthritis in mice (176) and to highly stimulate the production of TNF by macrophages (180). Importantly, these ACPAs presented highly mutated variable regions, which is suggestive of T cell help, with positive antigen selection through successive passages across germinal centers in secondary lymphoid organs, mucosae or, potentially, synovial ELS (176–178, 180–182). In fact, plasma cells expressing highly somatically mutated and multireactive ACPAs have also been recently identified in the synovial fluid of RA patients (181). These antibodies targeted distinct amino acid motifs and partially overlapping protein targets, supporting the hypothesis that multiple antigen encounters and selection processes occur sequentially, at different sites, such as the mucosae or the joints (181).

Further evidence directly implicating B cells in the initiation of RA was recently provided by a study demonstrating that the presence of dominant B cell receptor clones in peripheral blood of at-risk subjects is associated with a 6-fold increase in the risk of arthritis development (183). Moreover, as clinical disease ensued, these clones migrated to the synovial compartment, strongly implicating them as pathogenic (183).

#### Changes in immunoglobulin glycosylation

Another important aspect in the maturation of the RA immune response that has recently been unraveled, relates to the glycosylation profile of both the fragment crystallizable (Fc) and variable region of the ACPA molecule. It has been demonstrated that in established RA, up to more than 90% of IgG ACPAs are glycosylated in their variable domain, a much higher figure in comparison to polyclonal human IgG of healthy donors (15–25%) (146, 182, 184). Of note, the N-linked glycosylation site has been confirmed to be introduced during the process of somatic hypermutation (182, 185), which is in line with the studies demonstrating extensive mutation of ACPA IgG variable regions (176–178, 180–182), and with the absence of such changes in IgM ACPA (186).

Importantly, the extensive variable region N-glycosylation seems not to result from random accumulation of mutations, but rather reflects a competitive advantage for survival and differentiation of ACPA-positive B cell clones, allowing them to escape tolerance checkpoints and generate RA-specific autoimmunity (146, 182, 184, 185). The relevance of these findings for the development of RA has been most recently confirmed in two independent longitudinal studies, which have found that variable domain glycans are present in IgG ACPA up to 15 years before RA onset and their frequency increases progressively up until overt clinical disease (187, 188). Glycosylation of IgG ACPA variable region was higher in at-risk individuals who later developed RA (HR 6.07, 95% CI 1.46–25.2) (187). Further, it was associated with the presence of HLA-SE alleles, thereby suggesting a novel link with this genetic risk marker (188).

In contrast, the Fc tail glycosylation of serum IgG is reduced in patients with RA, as a result of the loss of terminal galactose and sialic acid residues (189–191). This hypoglycosylation profile is associated with a pro-inflammatory phenotype, as the IgG molecule more easily binds Fc receptors (FcRs) and activates complement, being correlated with disease activity and severity (146, 190, 192). Moreover, the Fc galactosylation and sialylation is lower in ACPA-IgG, when compared to total IgG, and in synovial fluid vs. serum ACPA-IgG (193). Notably, a decrease in galactosylated IgG and ACPA-IgG precedes the onset of RA by 3.5 years and 3 months, respectively (190, 191). This is paralleled by a rise in systemic inflammation, as assessed by ESR (191). Data coming from mice studies have implicated the interleukin (IL)-23-T helper (Th)17 axis (*via* IL-21 and IL-22) in the reduction of IgG Fc sialylation that will lead to arthritis (146). Whether a similar mechanism can be present in humans is an attractive, though unproven, hypothesis that would directly link Fc glycosylation with the evolution of autoimmunity to clinical disease.

#### Expansion of systemic inflammation

Finally, the amplification of the immune response that predates RA is also characterized by expanding systemic inflammation. Circulating cytokines (e.g., IL-1, IL-6, IL-12, tumor necrosis factor [TNF], interferon-γ, granulocyte–macrophage colony-stimulating factor), chemokines (e.g., monocyte chemotactic protein-1, macrophage inflammatory protein-1) and common markers of inflammation such as C-reactive protein have all been shown to increase progressively during the preclinical stages of RA, paralleling or even preceding the appearance and maturation of pathogenic autoantibodies (194–196). Such changes reflect the true systemic nature of the inflammatory mechanisms that will eventually culminate in overt clinical disease.

### Targeting of bone and joint

Despite remarkable progress in the comprehension of the disease pathogenesis, the mechanisms behind the transition from RA systemic autoimmunity, initiated at distant compartments like the mucosal surfaces, to localized and specific joint inflammation remain incompletely understood. The wealth of epidemiological data discussed above, showing a robust association of RF and ACPA with RA development, activity and severity, suggest that these autoantibodies are likely to be directly involved in the initiation of the arthritis that is characteristic of the disease. However, notwithstanding some major advances in recent years, this is still an area of ongoing controversy and a clear-cut pathogenic effect of RF/ACPA remains to be convincingly demonstrated and replicated (197).

A few important observations regarding the putative role of ACPAs and RF in joint targeting have been made. First, maternal transference of ACPAs and RF to the fetal circulation fails to provoke disease in the newborn (6). Second, ACPAs are multi-reactive (see above), can bind a number of citrullinated forms of proteins, which in their native form can be found in several tissues, and the relevant antigen target and tissue *in vivo* remains unknow (4–6, 197, 198). Third, ACPAs are, by themselves, insufficient to provoke synovitis, but can exacerbate and perpetuate an ongoing arthritic process (5, 6, 199). Under this model, an additional, perhaps preceding event, that causes an acute or subacute form of arthritis – a second *hit*, such as trauma, a viral infection or other unclear microvascular or neuroimmune factors – would be required to, in the presence of circulating ACPAs and/or RF, set in motion the pathogenic chain of events that will ultimately result in an autonomous, persistent, chronic synovitis (1, 6).

Nevertheless, it should be stressed that the pathogenicity of ACPAs has, to date, not been demonstrated in animal models. They have, in fact, been shown to be absent from common experimental arthritis models in mice and rats, such as collagen-induced arthritis (CIA) (200, 201), collagen antibody-induced arthritis (CAIA) (201), human TNF-transgenic mice (201, 202), adjuvant induced arthritis (201), and pristane-induced arthritis (PIA) (201, 203). Instead, what has been identified in such models, is the presence of measurable anti-CarP antibodies in mice with CIA and rats with CIA, PIA and AIA (200, 201). These arthritis models share the common feature of requiring the direct involvement of the adaptive immune system. This suggests that the tolerance to carbamylated antigens is breached early in the pathogenic process, unlike what is seen for citrullinated peptides, again questioning the pathogenicity of ACPAs. Another key aspect that is observed in these animal models, is the targeting of uncitrullinated forms of autoantigens widely recognized in RA, such as vimentin, histones, filaggrin or heterogeneous nuclear ribonucleoprotein (hnRNP)-A2 (also known as RA33) (107, 202, 203).

Autoantibodies against native (i.e., unmodified, uncitrullinated) proteins have been reported to be common in seropositive and seronegative RA. Among the most frequently described are FcV receptor (targeted by RF), PADs (most notably, PAD4), hnRNP-A2 (RA33) and (112, 114, 115, 204–207). The former two have been addressed above, while the latter has experienced a renewed interest in recent years. As previously mentioned, anti-RA33 antibodies have been identified in animal models of arthritis (202, 203). This antigen is overexpressed in the joints of human TNF-transgenic mice and PIA rats, and a specific humoral response targeting RA33, but not ACPAs, takes place in both preclinical and clinical phases of the disease. An important study by Konig et al. recently demonstrated a dual antibody response against both native and citrullinated forms of RA33 in the synovial compartment of patients with RA (205). The authors proposed an antigen-centered model, wherein an initial immunologic response would target RA33 in its unmodified form. According to this model, citrullination would only occur at a later stage, amplifying, but not starting, the immune response. This is demonstrated by the association of antibodies targeting citrullinated RA33 (i.e., ACPAs) with disease duration and erosive disease, in contrast with anti-RA33 antibodies, which were limited to early, mild, non-erosive RA. This transition from an anti-native to an ACPA response would depend on genetic (e.g., *HLA-DR* risk alleles) and environmental factors (e.g., smoking, infections, LTH). This is supported by studies demonstrating that the immune response seen in chronic bacterial infections, such as periodontitis or bronchiectasis, mainly targets non-citrullinated antigens in its earlier stages (130, 208). Future studies investigating this hypothesis are expected to provide further insight in the specific mechanisms involved in the targeting of rheumatoid joints.

#### Preclinical synovial inflammation

Prior to the emergence of clinically detectable arthritis, however, an earlier intermediate step is postulated to take place. This is when the first symptoms begin to appear, usually in the form of arthralgia (7). In this stage, most patients have evidence of ongoing autoimmunity (e.g., ACPA positivity) and start to experience what has been defined as clinically suspicious arthralgia (9). The reasons for these symptoms have been a matter of debate. In fact, evident imaging and histological synovitis were shown to be absent in subjects at high-risk of progressing to RA and could not discriminate which patients would develop the disease, suggesting that mechanisms other than synovial inflammation could explain patient symptoms (209, 210).

Nonetheless, there are indications that at least some degree of joint inflammation, not detectable by clinical examination, could be present before the onset of full-blown arthritis. In one of the studies addressing at-risk subjects, synovial infiltration by T cells was marginally associated with later development of arthritis (HR 2.8, 95% CI 0.9–9.1), particularly when circulating ACPAs were also positive (HR 3.8, 95% CI 1.3–11.3) (210). Moreover, the presence of CD8^+^ T cells in the synovium was strongly associated with ACPA positivity (OR 16.0, 95% CI 1.7–151.1), suggesting that specific joint citrullinated antigens may be targeted by the RA immune response (210). Synovial macrophage and T cell infiltration have also been demonstrated prior to the onset of arthritis in immunized monkeys and in clinically uninvolved joints of RA patients (211). It is thus possible that at least part of the symptoms experienced by patients during this phase, already reflects some degree of synovial inflammation, not clinically identifiable, that may be related to the presence of ACPAs and RF, closely preceding the installation of flagrant arthritis. This concept is supported by more recent studies reporting increased rates of subclinical synovial and bone inflammation defined by magnetic resonance imaging (212–214) and ultrasound (215, 216) in patients with clinically suspicious arthralgia. Of note, these changes were able to predict progression to RA in both ACPA-positive (215, 216) and ACPA-negative disease (214).

It seems apparent that, at least in the final stages of preclinical phase, targeting of the synovial compartment starts to occur. In this regard, ACPAs and RF have been proposed to play a pathogenic role (197). Recognition and binding of joint citrullinated antigens by ACPAs can lead to immune complex formation and activation of the complement cascade, that will result in recruitment of immune cells into the synovial compartment (217, 218). Incoming and resident cells, such as macrophages, mast cells, neutrophils and synovial fibroblasts, can then be activated by these immune complexes *via* interaction with surface FcγRs (and also TLRs) (197, 218–222). Cellular activation leads to the release of cytokines (e.g., TNF, IL-6) and other inflammatory and vasoactive mediators, enhancing and propagating the synovitis process (197, 218–222). Importantly, RF also seems to contribute significantly to this process, as it binds to synovial ACPA immune complexes and boosts their pro-inflammatory actions of complement activation and cytokine production (223–225). Moreover, the discussed ACPA Fc hypoglycosylation observed in RA and pre-RA patients further contributes to greatly increase the immune complex-induced activation of FcγR and complement, reinforcing the hypothesis that ACPAs are closely linked to synovitis initiation (146, 190, 191, 193). Overall, these studies support the working model in which the developing humoral response culminates, possibly following a predisposing insult, in the targeting of synovial citrullinated antigens by ACPAs and RF. These have, together and individually, enhanced ability to spark the inflammatory response typical of synovitis, which, albeit not clinically recognizable at first, becomes evident as the process advances.

#### Direct ACPA-osteoclast interaction

Even considering that pre-arthritis symptoms may be due to undetectable degrees of synovitis, the possibility still remains that other alternative pathways are in place. Accordingly, a new theory that directly implicates ACPAs – in an osteoclast-centered, synovitis-independent process – has been proposed in recent years, generating great controversy in the field (6, 8, 197). In this model, not only arthralgia but also early cortical bone loss, reported to be significantly increased in ACPA-positive pre-RA patients (226), could be the result of the interaction of ACPAs with key bone constituents. Osteoclast precursors have been shown to express citrullinating enzymes (i.e., PAD) and surface citrullinated proteins (e.g., vimentin, actin) that can be directly targeted by ACPAs (227–229). This induces the release of TNF and CXCL8 (i.e., IL-8), which promote osteoclast differentiation and activation in an autocrine fashion, leading to osteopenia and bone resorption *in vivo* (181, 227, 229). Notably, these processes were blocked by inhibition of PAD (229), CXCL8 (229), or TNF (227). These findings were initially replicated in a separate study (228), but the citrulline-binding specificity of the supposed ACPAs was subsequently not confirmed (230), suggesting that other mechanisms or technical reasons (e.g., antibody aggregation) might be involved (197, 231).

An alternative mechanism linking ACPAs and bone targeting was later proposed, as the interaction of immune complexes with FcγRs of pre-osteoclasts was demonstrated to promote osteoclastogenesis (232). This effect was dependent on IgG sialylation status, with desialylated (but not sialylated) immune complexes (and ACPAs) greatly stimulating osteoclast differentiation *in vitro* and *in vivo* (232). These findings are in accordance with the discussed increased pathogenicity of Fc hypoglycosylated ACPAs and total IgG reported in pre-RA and RA (190, 191), as well as with the observation that RA patients with lower IgG and ACPA sialylation had more bone loss (232). Although these studies lack unequivocal replication, the interaction of ACPAs – either in Fc-dependent or independent fashion – with developing osteoclasts represents an appealing explanation for both the targeting of bone and joints by the autoimmune response started in other distant compartments and the early bone loss observed in pre-RA patients, prior to the dawn of inflammation (6, 226).

In the second complementary part of this theory, ACPAs have also been proposed to have a direct link with joint pain in pre-RA, independently from synovitis (6, 233). The hypothesis proposed that osteoclast-bound ACPAs would induce the release of CXCL8, which could then bind and activate its receptors (CXCR1 and CXCR2) on nociceptive nerve terminals (233). In mice, this resulted in pain-like behavior that could be abolished by CXCR1/2 blockade (233). However, following the discovery that the antibodies used in this study were not ACPAs (230), this hypothesis has been questioned and, to date, could not be replicated (197). An alternative explanation could stem from CAIA models, in which antibodies against type II collagen are injected and bind to cartilage surface, occasioning immune complex deposition and florid synovial inflammation (234). In this model, mice experience pain-like behavior before the onset of arthritis and independently of complement activation, due to local immune complexes deposition which activate FcγRs expressed on sensory neurons (235). Interestingly, this mechanism could partially explain the results observed in the unconfirmed study (228), through cross-reactivity of polyclonal ACPAs that would recognize and bind citrullinated epitopes of type II collagen (235).

#### Immune response to joint targets

Finally, a few other pathways have been suggested to explain joint targeting in RA, mainly based on data arising from animal models and lacking substantial epidemiological support (1, 5, 6). These include the immune response to unmodified forms of ubiquitous enzymes such as glucose-6-phosphate isomerase (236) or joint-specific cartilage constituents (e.g., type II collagen) (237). While the former has been found not to be common or specific for RA (238), the latter has been one of the most studied and has recently gained renewed attention (237, 239). Anti-type II collagen antibodies have been found to be present in a subgroup of RA patients (3–27%), particularly during early disease, and may be associated with a particular phenotype of marked acute inflammation in a similar fashion to what is observed in CAIA and CIA experimental models (237, 240). These antibodies can lead to arthritis *via* immune-complex deposition and are also believed to have direct damaging effects in the cartilage (237). More recently, the relevance of type II collagen citrullination was proposed, as citrullinated forms of type II collagen were identified in the synovial fluid of RA patients (239) and were targeted by cross-reacting circulating ACPAs in 17–27% of patients (241). Additionally, ACPAs highly specific to citrullinated type II collagen have been demonstrated to be pathogenic in mice (239) and to be present in about a third of RA patients, linking ACPA and cartilage-targeting pathways (242). Whether, as proposed above, these ACPAs against citrullinated type II collagen are the result of the evolving initial immune response targeting native cartilage constituents, is a possibility that deserves further exploration.

### Disease mechanisms in seronegative RA

The events described above are applicable to seropositive disease, that is, where ACPA (and usually also RF) are present in circulation. This is the major RA subtype, which accounts for 70–80% of patients, both in the context of research and daily practice. Substantially less is known about the mechanisms involved in the pathogenesis of the seronegative subset of disease, that is, that without either ACPA or RF (243). It is important to address a few aspects concerning the definition and characteristics of this patient population. Although seronegative RA has long been recognized as a distinct clinical entity, there is still debate as to whether this really is the case. While some argue that these patients have a phenotype compatible with RA, but present different etiological and pathogenic pathways, others regard this classification as the result of unmeasured/unknown circulating autoantibodies (both ACPAs and non-ACPAs) in “true” RA patients. Others, still, consider this an heterogeneous population, possibly including patients who were misclassified or will evolve into other diagnoses over time (e.g., spondyloarthritis) (244). Although the majority of the driving forces behind seronegative RA are undetermined, some progress has been made over the last decade. Evidence that can inform in this regard is mostly indirect, but a few points can be made (summarized in Table 2).

**Table 2:** Essential aspects differentiating seropositive and seronegative RA.

**Key topics**	**Supporting data**
Mostly distinct genetic background (partially overlapping)	• Risk alleles (HLA and non-HLA) differentially associated with each subtype • Different susceptibility genes (e.g., *CLYBL, PRL, NFIA* for seronegative RA) • Some shared risk alleles with weaker association in seronegative RA • Lower heritability in seronegative RA (?)
Unknown/unmeasured autoantibodies (in seronegative RA)	• ACPA fine-specificities (30% of ACPA-/RF- patients) IgG/IgA RF (9%) • Anti-CarP (8–11%; not associated with SE, *PTNP22* or smoking; present in pre-RA) • AAPAs (30–40%) • Other: anti-PAD4 (2-19%), anti-progranulin (21%), anti-PTX3 (28%), and anti-DUSP11 (32%)
Different environmental risk factors	• Smoking and silica exposure strongly associated with seropositive, but not seronegative RA • Discrepant associations with alcohol, coffee, obesity, depression, hormonal changes • Shared risk factors (physical workload, stressful life events) have different magnitude
Distinct pathogenic mechanisms in seronegative RA	• Enriched STAT3 and IL-6 signature • Different cytokine profile: **↑**IL-10, IL-8, IL-13/**↓**IL-1ß, IL-5, IL-15, IL-17A, eotaxin • Increased NK cells • Lower histological synovitis burden: **↓**B and T cells, ELS, lympho-myeloid pathotype/**↑**fibrosis, pauci-immune pathotype • Broader repertoire of synovial T cells
Clinical differences	• Seronegative arthralgia/pre-RA: **↓**symptom duration **/** **↑**lower extremities, tender joint counts, time to arthritis development • Seronegative RA less severe/better prognosis (?) – not confirmed in bDMARDs era • Possible reclassification of seronegative patients over time (e.g., spondyloarthritis)

AAPA, anti-acetylated peptide antibodies; ACPA, anti-citrullinated protein antibodies; Anti-CarP, anti-carbamylated protein antibodies; bDMARDs, biologic disease-modifying anti-rheumatic drugs; ELS, ectopic lymphoid structures; PAD, peptidyl-arginine deiminase; PPAD, Porphyromonas gingivalis peptidyl-arginine deiminase; RF, rheumatoid factor.

First, there is evidence that ACPA-positive and ACPA-negative RA have mostly distinct genetic backgrounds (243). This has been suggested by several studies, with risk alleles differentially associated with each subtype being identified both within and outside the HLA region (245–252). The latter included some genes that are also associated with seropositive RA (e.g., *TNFAIP3, PTPN22*), as well as a few novel susceptibility genes (e.g., *CLYBL, PRL, NFIA*) (245, 250). Also, the strength of the associations seen for shared risk alleles is different between both subsets in a number of studies, with lower effect sizes in ACPA-negative RA (245–250). Likewise, differences were even noted in specific amino acid residues of the HLA molecule for both disease subsets (246). These findings are in accordance with the recently reported lower heritability in seronegative RA (~20%), in comparison with seropositive disease (50%) (253). However, other studies have suggested that genetic risk of both subsets greatly overlaps (254–256). Therefore, the current view is that ACPA-positive and ACPA-negative RA do share some genetic risk markers, but there are substantial differences between these two subsets, with several susceptibility genes more strongly associated with one or another subtype.

Second, another possibility is that patients classified as seronegative have other unknown or unmeasured circulating autoantibodies. A recent study showed that many classically-defined seronegative patients had ACPA fine-specificities (30%), IgA/IgG RF (9.4%) or anti-CarP antibodies (16%), thus underlining that a considerable proportion of “seronegative” patents are wrongly classified as such (257). Anti-CarP antibodies had been originally identified also in 16% of ACPA-negative patients, who had a more severe disease course than those negative for both anti-CarP and ACPA (258). The presence of these autoantibodies in ACPA-negative patients was replicated in other cohorts (8–14%), and was not associated with *HLA-DRB1* SE alleles, *PTPN22* or smoking, suggesting distinct biological mechanisms in comparison with ACPA-positive RA (259). Anti-CarP antibodies have now been shown to be present years before the onset of clinical disease, in around 10–11% of ACPA/RF-negative pre-RA patients, and predict incident RA independently of ACPA (16, 260, 261). These findings suggest that anti-CarP is involved in the pathogenesis of RA, including of ACPA/RF-negative disease. Anti-acetylated peptide antibodies (AAPA) are another important class of non-ACPA autoantibodies that was recently described in 30–40% of patients with early or established seronegative RA (262, 263). Further, diagnostic accuracy was greatly increased when positivity against more than one acetylated peptide was observed, with AAPAs against three acetylated peptides being 100% specific for RA (262). Together, ACPAs, anti-CarP and AAPAs may be classified as anti-modified-protein antibodies (AMPAs) (264). The various AMPAs may present cross-reactivity between each other to different extents, i.e., to one, two or all three of them (264–267). In patients with new-onset RA, triple reactivity has even been shown to be associated with higher rates of radiographic progression at 12 months than single reactivity (265). Finally, novel antibodies were recently identified in a substantial proportion of seronegative patients, such as anti-PAD4 (2–19%) (96, 109, 111), anti-progranulin (21%), anti-PTX3 (28%), and anti-DUSP11 (32%) antibodies, further expanding the comprehension of ACPA-negative disease (268, 269).

Third, an additional argument in favor of a distinct pathogenesis between ACPA-positive and ACPA-negative RA is supported by the differences observed in environmental risk factors for both subsets (3). Some of the major risk factors for RA, such as smoking (270) or silica (46) exposure, are strongly associated with seropositive, but not seronegative RA (6). Similarly discrepant findings have been reported for variables as different as alcohol consumption (271), coffee intake (272), obesity (273, 274), depression (275) or hormonal changes (276). Of note, as for genetic background, some environmental risk factors, such as physical workload (277) or stressful life events (278), associate with both subtypes, but on a different magnitude. Again, these data are indicative that the mechanisms occurring in the preclinical phases of seropositive and seronegative RA are at least partially distinct.

Fourth, although significantly less is known about the mechanisms leading to, and driving, seronegative RA, some evidence has emerged over the last decade. Circulating CD4^+^ T cells of untreated undifferentiated arthritis patients have been shown to have a transcriptional signature enriched in STAT3-inducible genes, which predicts the evolution to RA, especially to seronegative disease (279). This gene expression pattern was correlated with IL-6 levels, which were higher in patients with ACPA-negative RA (279). IL-6 signals mainly *via* STAT3, and an IL-6-driven STAT3 signaling pathway in CD4^+^ T cells has been identified in the early phases of seronegative RA (280). These pairs well with the strong genetic association of ACPA-negative RA with *ANKRD5*, a gene greatly expressed by CD4^+^ T cells, which lies close to the locus encoding glycoprotein 130 (gp130), a signal transducing component of the functional receptor complexes for IL-6 (249, 252). In addition to a putative prominent role of IL-6 in seronegative RA, another study demonstrated a distinct cytokine profile of patients with early ACPA/RF-negative disease, with increased IL-10 and decreased IL-1ß, IL-15 and eotaxin in levels in comparison with patients with seropositive RA (281). Further, IL-5 and IL-17A were not altered in seronegative arthralgia patients (contrary to RF-positive disease), whereas an increase in IL-8 and IL-13 could be seen in this population (194). The former could directly induce pain by binding to nociceptors, while the latter promotes synovial hyperplasia and is secreted by CD56^bright^ natural killer (NK) cells, which are increased in seronegative RA and also produce IL-10 (282, 283). Thus, however slim and lacking replication, these data suggest that distinctive mechanisms may underlie the preclinical phases of seronegative RA.

Additional evidence stems from the comparison of synovial tissue features of ACPA-positive and ACPA-negative patients. Patients with established seronegative RA were reported to have a lower synovitis burden, translated by a lower infiltration of B and T cells (especially CD8^+^), less lymphoid aggregates (and corresponding lympho-myeloid pathotype), more fibrosis, and a higher representation of a pauci-immune pathotype (284–286). In addition, synovial T cells have a more restricted repertoire in ACPA-positive, in comparison with ACPA-negative patients, suggesting a more antigen-driven pathway in seropositive disease (287). Interestingly, CD68^+^ sublining macrophages have similar distributions in both subsets (284–286).

Fifth, a few words are warranted for the clinical aspects of seronegative RA. A recent study has demonstrated that the preclinical phases leading to RA are different in ACPA-positive and ACPA-negative patients (288). Patients with seronegative arthralgia had more often involvement of the lower extremities, higher tender joint counts, shorter symptom duration, and more difficulty making a fist. However, after presenting with arthralgia, ACPA-negative patients took longer to develop arthritis, thus suggesting that seropositive and seronegative disease develop differently. In terms of disease course, RF/ACPA-negative RA has traditionally been regarded as less severe, and indeed, seropositivity is a recognized poor prognostic factor (289–291). However, studies of patients treated in the novel biologic treatment era have demonstrated that patients with seronegative RA have higher baseline disease activity, disability, patient reported outcomes and erosive burden (292, 293). Further, although these patients responded well to DMARD therapy, the treatment intensity and requirement was similar to RF/ACPA-positive patients, and serological status was not associated with remission (293). Likewise, functional, social and occupational impairment were similar, contradicting the view of seronegative RA as a less impacting disease (292). Finally, it should be acknowledged that the group of patients classified as seronegative RA may include other diseases that present in a RA-like fashion and develop manifestations of other conditions later in the disease course. In fact, recent studies demonstrated that a significant proportion of seronegative patients are reclassified as having another diagnosis over long-term follow-up, most notably spondyloarthritis (244, 294).

In summary, the pathogenesis of seronegative RA is still mostly unknown. The limited available data suggest that the mechanisms leading up to the onset of clinically evident disease are at least partially different from those described for ACPA-positive RA. In addition, novel autoantibodies are being described, which could lead to a significant reduction in the pool of real ‘seronegative’ patients with RA. Much more so, when considering that many of them will eventually be reclassified as having another disease in the future. This is an expanding field, where our understanding of the true nature and driving forces behind seronegative RA is likely to increase substantially over the next years.

## Discussion

Tremendous progress has been made over the last decades in the understanding of early pathological events that occur before the onset of RA. One of the major insights that has since arisen is the concept that the initial immune disturbance mechanisms take place outside the joint compartment. Indeed, albeit being mostly localized to the joints, RA is truly a systemic disease even since its primordial stages. In this regard, mucosal tissue plays with a high probability a crucial role, as the interface between self and environment where the initial immunologic response to altered antigens commence. The lungs are the foremost site driving the process that will culminate in breach of tolerance and triggering of autoimmunity, closely followed by the gingival and intestinal mucosae.

What is unique in this theoretical model is that it clearly explains the close connection between environment (e.g., smoking) and host (e.g., HLA-SE) in determining the development and onset of disease. It provides a basis for the strong epidemiological observations that pinpoint specific risk factors for incident RA. Further, it strengthens the recommendations made for at-risk subjects, such as quitting smoking, maintaining good oral and periodontal health, and following a balanced diet, maintaining a low body mass index.

Attesting to the systemic nature of RA, the adaptive immune system will likely drive the amplification of the immune response that follows mucosal loss of tolerance, expanding it to secondary lymphoid organs. Herein, APCs, T cells, B cells, and plasma cells, closely interact, maturing the immune response over time, and resulting in a broader and more specific antibody repertoire. This is paralleled by modifications in the glycosylation status of immunoglobulins, including ACPA and RF, that increases their pathogenic and inflammatory potential, as well as a steady overall rise in systemic inflammatory markers.

Finally, the inflammatory response reaches the joint, probably prior to the advent of clinical symptoms. Circulating ACPAs recognize and bind synovial citrullinated antigens, activating an immune complex-driven local inflammatory response that is further amplified by RF. Other attractive mechanisms including ACPA-induced activation and differentiation of osteoclasts have been proposed and deserve further study. Once started, the synovitis process evolves into clinically recognizable arthritis.

The better comprehension of these preclinical pathophysiological events allows for an overall comprehension of RA as a complex systemic disease. It facilitates the improvement of prediction models and biomarkers, particularly useful for at-risk subjects (295); it contributes to the identification of key mediators of disease, which can be targeted by novel therapeutic agents (296); and it can ultimately revolutionize the paradigm of RA care, by directly interfering in the early preclinical stages of the disease with lifestyle modifications and specific therapies that can halt, or at least delay, the ongoing pathogenic immune response (297). These ideas have recently been tested in several observational studies and clinical trials (Table 3). In fact, the PRAIRI study provided proof-of-concept that such a strategy could be useful, by demonstrating that a single infusion of B cell-depleting rituximab delayed the onset of arthritis in at-risk individuals (302). The recently reported results of ARIAA study were also in line with this, wherein targeting T cell co-stimulation with abatacept for 6 months significantly decreased subclinical joint inflammation and the incidence of new-onset arthritis in ACPA+ subjects with arthralgia and imaging evidence of joint inflammation (306). Other studies are investigating a similar effect with treatments such as methotrexate or hydroxychloroquine (Table 3) (304, 305, 308).

**Table 3:** Summary of studies investigating lifestyle and pharmacological interventions in preclinical RA.

**Study name, type, reference**	**Population**	**Intervention**	**Comparator**	**Outcome**	**Follow-up time**	**Results**
PRE-RA, Unblinded RCT, Sparks 2018 (298)	FDR of RA patients (*n =* 238)	Web-based educational tool for RA risk factor education, with tailored advice	Standard RA education	Motivation for changing RA risk-related behaviors	6 months	Increased motivation to improve RA risk behaviors (RR 1.23, 95% CI 1.01–1.51)
SOS, POS, Maglio 2020 (299)	Obese subjects without RA (*n =* 4,036)	Bariatric surgery	Conventional treatment, matched on day of surgery	Diagnosis of incident RA^a^	Median 21 years (0–29)	Similar RA (or seropositive RA) incidence (2.3% vs. 2.2%, *p =* 0.88; HR 0.92, 95% CI 0.59–1.46)
NHS, POS, Liu 2019 (300)	Female nurses without RA or other CTDs (*n =* 230, 732)	Smoking cessation	Smoking continuation	Diagnosis of incident RA	6, 037, 151 person-years	Decreased trend for incident RA with increasing years since cessation (*p =* 0.009)
ISRCTN73232918, double-blind RCT, Bos 2010 (301)	ACPA and/or RF-positive arthralgia patients (*n =* 83)	IM 100 mg dexamethasone (0 and 6 months)	IM Placebo (0 and 6 months)	≥50% reduction of ACPA and/or RF levels. Arthritis development	Median 26 months (IQR 21–37)	Similar arthritis development (HR 1.1, 95% CI 0.4–2.8) and ≥50% reduction ACPA/RF levels, but significantly higher decreases in intervention.
PRAIRI, double-blind RCT, Gerlag 2019 (302)	ACPA+ and RF+ arthralgia patients, no clinical arthritis, CRP>0.6 mg/L *or* US/MRI synovitis (*n =* 81)	IV Rituximab 1g + 100 mg methylprednisolone	IV Placebo + 100 mg methylprednisolone	Time to development of clinical arthritis (≥1 swollen and tender joint)	Median 29 months (IQR 14–40)	12-month delay in arthritis development; non-significant risk decrease of arthritis development (HR 0.45, 95% CI 0.15–1.32)
STAPRA, double-blind RCT, van Boheemen 2021 (303)	Arthralgia patients with ACPA≥3x ULN *or* ACPA+ and RF+, no clinical arthritis (*n =* 62)	Atorvastatin 40 mg daily	Placebo daily	Development of clinical arthritis (≥1 swollen joint)	Median 14 months (IQR 6–35)	Similar rates in arthritis development (HR 1.40, 95% CI 0.50–3.95)^b^
APIPPRA, double-blind RCT, Al-Laith 2019 (304)	Patients with small joint inflammatory arthralgia, with ACPA≥3x ULN *or* ACPA+ and RF+, no clinical arthritis (target *n =* 206)	Abatacept 125 mg sc weekly	Placebo sc weekly	Time to development of clinical synovitis ≥3 joints or RA	52 weeks	Recruitment completed, results not available
StopRA, double-blind RCT, NIAID (305)	Subjects with raised anti-CCP3 ≥40 units, without clinical arthritis (target *n =* 200)	Hydroxychloroquine 200–400 mg daily	Placebo daily	Development of RA	36 months	Recruitment closed at *n =* 144, follow-up ongoing
ARIAA, double-blind RCT, Rech 2021 (306)	ACPA+ subjects with arthralgia, MRI synovitis *or* osteitis of dominant hand (*n =* 100)	Abatacept 125 mg sc weekly	Placebo sc weekly	Improvement of MRI synovitis or osteitis of dominant hand. Arthritis development	6 months	Greater improvement in MRI (61% vs. 31%, *p =* 0.004). Less incident arthritis (35% vs. 8%, *p =* 0.003).
TREAT-EARLIER, double-blind RCT Krijbolder 2022 (307)	CSA, no arthritis, MRI synovitis *or* osteitis of unilateral wrist, hand, foot (*n =* 236)	IM methylprednisolone 120 mg (x1) + methotrexate 25mg po weekly (12 months)	IM placebo (x1) + placebo po weekly (12 months)	RA or arthritis (SJC66 ≥2) development. Patient-reported function, symptoms and work productivity.	24 months	Similar rates in RA or arthritis development (HR 0.81, 95% CI 0.45–1.48), but sustained improvement in function, pain, morning stiffness, presenteeism and MRI-detected joint inflammation (all p <0.01)

a *Primary outcome of main study was mortality. Incident RA was not predefined endpoint*.

b *Trial prematurely stopped due to low inclusion rate*.

*CI, confidence interval; CSA, clinically suspect arthralgia; CTDs, connective tissue diseases; DMARD, disease-modifying anti-rheumatic drug; HR, hazard ratio; IM, intramuscular; IQR, interquartile range; IV, intravenous; MRI, magnetic resonance imaging; po, per os; POS, prospective observational study; RCT, randomized controlled trial; RR, risk ratio; sc, subcutaneous; SJC66, swollen joint count 66 joints*.

In this regard, it should be stressed that the designation of *at-risk* individuals has multiple definitions across studies. Such examples include first-degree relatives of RA patients, ACPA+ asymptomatic subjects, seronegative patients with CSA, patients with subclinical imaging synovitis, to name only a few. These subject groups may or may not be analyzed together or in different combinations, potentially diluting or scattering important signals arising from studies, translating independent mechanistic pathways. Thus, terminology standardization according to published guidelines is key in order to allow robust comparison between studies (7). In addition, types and timing of therapeutic or preventive interventions also vary, as do outcomes of interest. To tackle these issues, a data-driven consensus statement was recently published, providing additional guidance for research studies focusing on this population (309).

In conclusion, the pathogenesis of RA initiates well before the onset of clinical symptoms. In line with the complex nature of the disease, and the multitude of etiological risk factors, intricate pathogenic mechanisms take place both outside and within the joints, evolving over time until clinical disease becomes apparent. Future developments in the field are likely to provide new insightful data, potentially leading to a novel era of ever-earlier treatment institution, arresting disease progression and aiming at prevention of RA.

## Author contributions

VCR and JEF have contributed to study conception and design, collected and analyzed the data, and drafted the manuscript. All authors have critically reviewed the manuscript for important intellectual content, have read, and approved its final version.

## Funding

VCR work was funded by Fundação para a Ciência e Tecnologia (Interno Doutorando Bursary reference SFRH/SINTD/95030/2013); European League Against Rheumatism (EULAR Scientific Training Bursary 2014); and Sociedade Portuguesa de Reumatologia (Fundo de Apoio à Investigação da SPR 2015 & 2016 to VCR).

## Conflict of interest

The authors declare that the research was conducted in the absence of any commercial or financial relationships that could be construed as a potential conflict of interest.

## Publisher's note

All claims expressed in this article are solely those of the authors and do not necessarily represent those of their affiliated organizations, or those of the publisher, the editors and the reviewers. Any product that may be evaluated in this article, or claim that may be made by its manufacturer, is not guaranteed or endorsed by the publisher.
